# A Bibliometric Analysis of the Literature on Norovirus Disease from 1991–2021

**DOI:** 10.3390/ijerph19052508

**Published:** 2022-02-22

**Authors:** Ropo E. Ogunsakin, Oluwakemi Ebenezer, Themba G. Ginindza

**Affiliations:** 1 Discipline of Public Health Medicine, School of Nursing & Public Health, College of Health Sciences, University of KwaZulu-Natal, Private Bag X54001, Durban 4000, South Africa; ginindza@ukzn.ac.za; 2 Department of Chemistry, Faculty of Natural Sciences, Mangosuthu University of Technology, Durban 4000, South Africa; re.korede@gmail.com; 3 Cancer & Infectious Diseases Epidemiology Research Unit (CIDERU), College of Health Sciences, University of KwaZulu-Natal, Private Bag X54001, Durban 4000, South Africa

**Keywords:** norovirus, publication trend, collaboration, Web of Science, citation classics, bibliometrics

## Abstract

Norovirus (NoV) is one of the oldest recognized diseases and the leading causal pathogen for acute gastroenteritis (AGE) worldwide. Though numerous studies have been reported on NoV disease, limited research has explored the publication trends in this area. As a result, the objective of this work was to fill the void by conducting a bibliometric study in publication trends on NoV studies as well as discovering the hotspots. The Web of Science central assemblage database was hunted for publications from 1991 to 2021 with “norovirus” in the heading. Microsoft Excel 2016, VOSviewer, R Bibliometrix, and Biblioshiny packages were deployed for the statistical analysis of published research articles. A total of 6021 published documents were identified in the Web of Science database for this thirty-year study period (1991–2021). The analyses disclosed that the *Journal of Medical Virology* was the leading journal in publications on norovirus studies with a total of 215 published articles, the *Journal of Virology* was the most cited document with 11,185 total citations. The United States of America (USA) has the most significant productivity in norovirus publications and is the leading country with the highest international collaboration. Analysis of top germane authors discovered that X. Jiang (135) and J. Vinje (119) were the two top relevant authors of norovirus publications. The commonly recognized funders were US and EU-based, with the US emerging as a top funder. This study reveals trends in scientific findings and academic collaborations and serves as a leading-edge model to reveal trends in global research in the field of norovirus research. This study points out the progress status and trends on NoV research. It can help researchers in the medical profession obtain a comprehensive understanding of the state of the art of NoV. It also has reference values for the research and application of the NoV visualization methods. Further, the research map on AGE obtained by our analysis is expected to help researchers efficiently and effectively explore the NoV field.

## 1. Background

Norovirus (NoV) was first discovered in 1968 [1] and has caused broadband of diseases [2]. The virus belongs to the family of Caliciviridae. NoV is single-stranded, small non-enveloped RNA [3,4]. The first track-down *Norovirus* was linked with a human eruption of gastroenteritis in Norwalk, Ohio. It was discovered from clinical specimens during the study of a gastroenteritis outbreak among pupils in Norwalk, Ohio, and gave rise to the name *Norwalk-like viruses* (NV) in 1968 [1] and later called *Norovirus*. The strain from the sample was discovered in 1972 and was the first virus identified to cause gastroenteritis in humans. The virus has also been identified in animals, and there is a substantial universal disease problem triggered by norovirus infection. In addition, noroviruses were classified into six genogroups (GI to GVI), with the seventh genogroup (GVII), which has been recently suggested [5,6,7].

Meanwhile, GI, GII, and GIV strains occur in humans, and GIII and GV strains occur in cows and mice, separately [8]. The GII.4 genotype is the source of most norovirus occurrences in individuals. The human norovirus (HuNoV) pandemics in previous years, specifically, 1996–1997, 2002, 2004, 2006, 2009, and 2012–2013 in the United States, Netherlands, Australia, and the Asia continent, have been induced by the onset and rapid spread of GII.4 [8].

Studies on the epidemiological characteristics of a gastroenteritis outbreak caused by NoV include transmission of pathogens through food-borne exposure, food handling, person-to-person contact, and contaminated water [9,10,11,12,13,14,15]. Many pieces of literature have also reported that NoV is one of the leading causes of acute gastroenteritis in children less than 5 years of age [16,17,18]. At present, there are no vaccines or medicines to treat norovirus infection. The lack of a reactive cell culture system for NoV replication has hurt the development of NoV problem-solving methods. Thus, studies on NoV are significant. However, no bibliometric study has been performed to the best of our ability to evaluate published patterns on NoV. Currently, bibliometric analysis increasingly plays a significant part in combining previous studies’ outcomes to effectively advance the current information to develop a line of study and result in evidence-oriented understanding.

Moreover, several studies have been researching norovirus diseases globally since its inception. Based on the search emanated from this study, more than 6000 documents associated with NoV disease have been printed in the literature (Web of Science). Studies stemming from such investigation have to aid in information about the informed nature of such research and discover breaches. Similarly, investigators need to dedicate a substantial volume of time to reading and detecting appropriate work in interrelated disciplines considering a wide range of studies, irregular superiority of scientific research papers, a considerable amount of data, and the changes between norovirus and evolving viruses. Consequently, it is imperative to categorize substantial, active, and evocative evidence from massive databases to assist the scientific investigation. Hence, this is for investigators to establish innovative outlines of investigation and assist policymakers in appraising and recommending methods to improve the study holes. An approach of statistical analysis termed bibliometrics is deployed to evaluate the essential evolving inclinations and features of a specified study subject built on the printed studies [19].

Bibliometrics and visualization have been conveyed as an essential way to detect evolving occurrences of infectious disease, and it is an imperative technique of scientific research evaluation. This observation is correct considering the situation of the present age, where multiple amounts of information is being exchanged between healthcare providers [20,21]. Similarly, bibliometrics is usually used in various disciplines to appraise scientific research quantitatively and qualitatively [22]. Then, to systematically divulge associates within the norovirus family, our study deployed bibliometrics and visualization approaches to analyze norovirus-linked publications and citations, countries, and author impact. Based on the evidence gathered through the research, and to the best of our knowledge, this is the first bibliometric study on trends in NoV research related to epidemiological research in the world; 4303 published articles were repossessed and statistically analyzed in the present bibliometric analysis. This study’s findings inform the NoV studies’ hot spots and can benefit the development of a national and institutional research strategy. Additionally, the resulting data or evidence from the visualizations can be used to study the scientific history of investigation outputs in a definite discipline and recognize the potential future investigation path and prospects for collaboration [23].

## 2. Search Strategy and Research Methodology

### 2.1. Search Strategy

**Figure 1:**
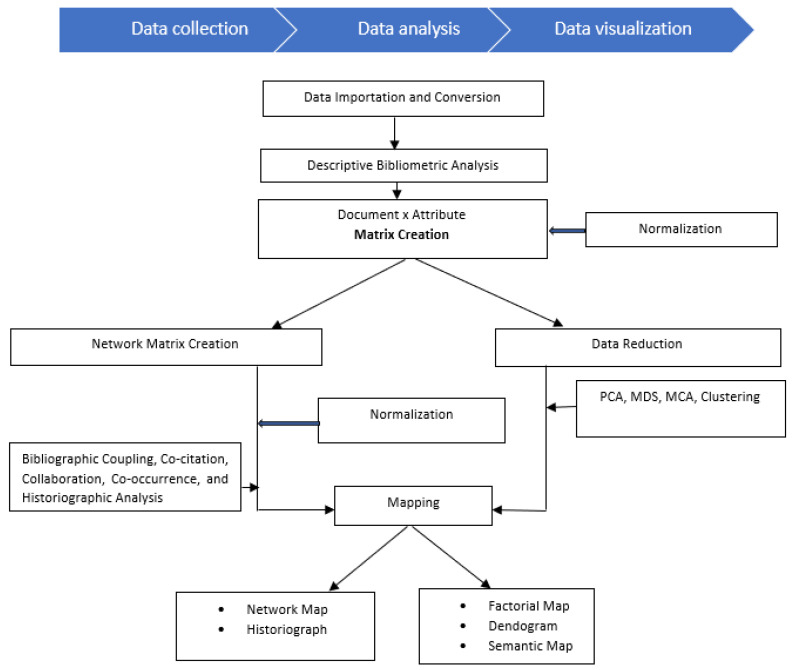
Bibliometrics processing of the information and suggested science mapping.

A pilot search was conducted using the world’s largest and most comprehensive database of information resources, i.e., Web of Science (WoS). Today, the Web of Science is the most comprehensive and inclusive collection of information materials globally. It contains over 11,000 authoritative academic journals that significantly impact the natural sciences, engineering, biomedicine, etc. We used the Web of Science^TM^ core collection within the WoS database as a data source for this research. The document type is the article, the search method is a title search, and the language is all languages. The keyword “norovirus disease” was used as a topic term to search documents that contained this word in the title or keyword or abstract of the documents published from 1991 to 2021. In some cases, we also used the keyword “Norwalk disease.” A total of 6021 documents from 1991 to 2021 were found from the database, with the following types of documents: article (4934; 81.95%), review (542; 9.00%), conference (268; 4.45%), letter (95; 1.58%), editorial (90; 1.49%), note (11; 0.18%), book chapter (11; 0.18%), retracted (6; 0.09%), reprint (9; 0.15%), others (55; 0.91%). Hence, we chose articles for the final analysis because articles contain a description of complete research and results. Data around these articles and the total annual citations for each article were downloaded. Comprehensive document lists were exported as BibTeX. Finally, to analyze the data extracted and the needed results, it is essential to consider five vital phases that permit quantitative and qualitative analysis used for a bibliometric analysis (Figure 1). As a result, the fundamental objective of this research is to link studies from various years, countries, and journals to the same research parameters.

### 2.2. Research Methodology

Bibliometrics analyses permit us to find an unfathomable perception from a moderately comprehensive study. Bibliometrics tool in R package is intended to be used in quantitative scientometrics and informetrics [24]. The tool offered various measures for importing bibliographic data from Scopus, Clarivate Analytics Web of Science, Digital Science Dimensions, Cochrane Library, Lens, and RISmed PubMed/MedLine. Further, the bibliometric packages permit classifying and analyzing vast quantities of historical data from research steered over a definite period to acquire metadata from the database. After importing the documents, data pre-processing and bibliometric analysis on the sources, authors, authors’ affiliation, and authors’ countries were explored. Besides, building networks for co-citation, clustering by coupling, scientific collaboration, and co-word analyses were performed, and the obtained graphs were visualized. The VOSviewer package of R language was applied to visualize the collaboration network of high-producing countries and authors in NoV disease research [25]. The command vos.path = ““, type = “VOSviewer”, size = T, delete; multiple = T has been used to call the VOSviewer software application and generate cooperative maps for both countries and authors. The visualization maps were created with the help of VOSviewer 1.6.17 (Centre for Science and Technology Studies, Leiden University) to analyze and visualize any relationships among authors, countries, and the terms used in the papers [25]. Microsoft Excel performed time trend analyses (a linear graph, pie chart, and bar chart).

Similarly, we employed a data mining approach for the analysis of keywords. Data mining in bibliometrics of the frequency of concurrent occurrence of two research papers’ keywords was performed through statistical software package Biblioshiny to abridge the intricate keyword network association into moderately small assemblies. The data mining approach deployed is the multiple correspondence analysis (MCA) and hierarchical clustering approach. Multiple correspondence analysis is a recent statistical technique gaining momentum in the health-related field. This approach compresses a massive amount of data into low dimensions to form two-dimensional graphics to mirror their resemblance. In the case of this study, the keywords that are nearer to the center of the map and dispersed more intensely are the keywords that have received added attention during recent years, while those that are more consistently spread are linked to less often discussed research topics. Additionally, the second data mining approach used is hierarchical clustering (hierarchical cluster analysis). Hierarchical clustering is an algorithm that groups similar objects into groups called clusters. The endpoint is a set of clusters, where each cluster is distinct from the other cluster, and the objects within each cluster are broadly similar to each other. Meanwhile, cluster analysis in bibliometrics is built on the frequency of concurrent occurrence of two keywords, using a data mining approach to abridge the intricate keyword network association into numerous relatively small groups.

## 3. Results and Discussions

**Figure 2:**
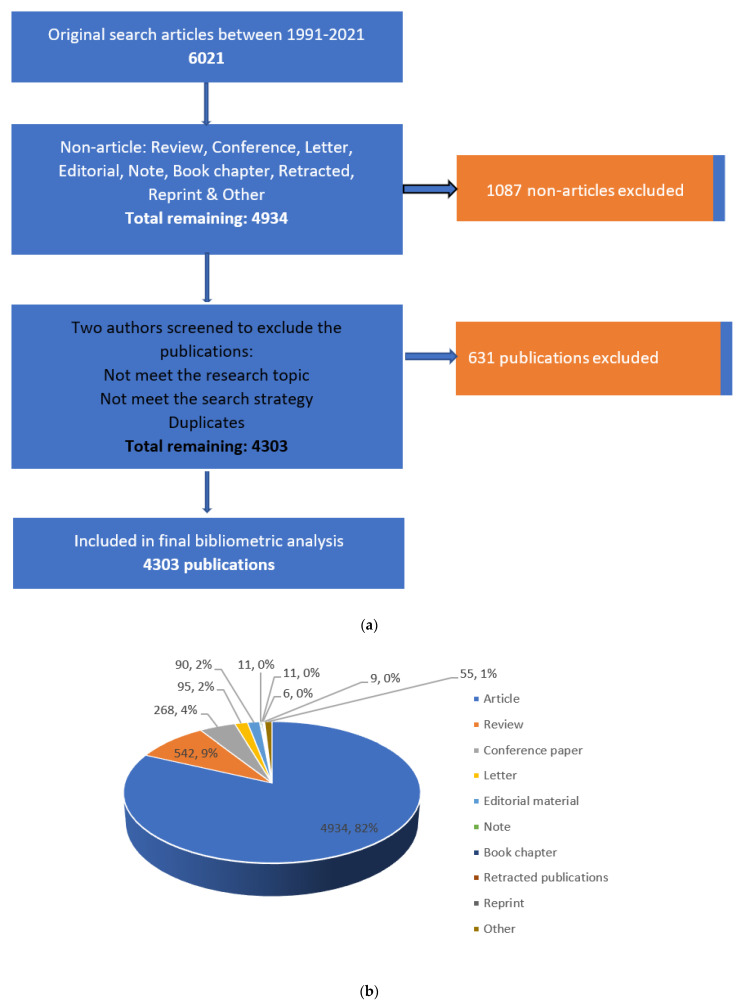
(**a**) Data processing flow chart for norovirus disease; (**b**) Graphical representation of the article extracted.

Exported documents are restricted to articles published in English. This was competent by restricting the results to English only during the research. Six thousand and twenty-one studies (6021) were identified based on the literature search, of which 631 duplicate entries were identified and removed. In addition, 1087 non-articles were removed. Finally, 4303 relevant papers were eligible for the final bibliometric analysis (Figure 2a). Similarly, as can be seen in the pie chart, we observed that a larger proportion of the extracted publications are articles, representing 82% (4934) of the total; this is followed by reviews, which, at 8%, forms nearly half of the remaining portion. Further categories of documents, in descending direction, were review (n = 542, 9%), conference paper (n = 268, 4%), letter (n = 95, 2%), editorial material (n = 90, 2%), other (n = 55, 1%), and the remaining are less than 1%, respectively (Figure 2b).

**Figure 3:**
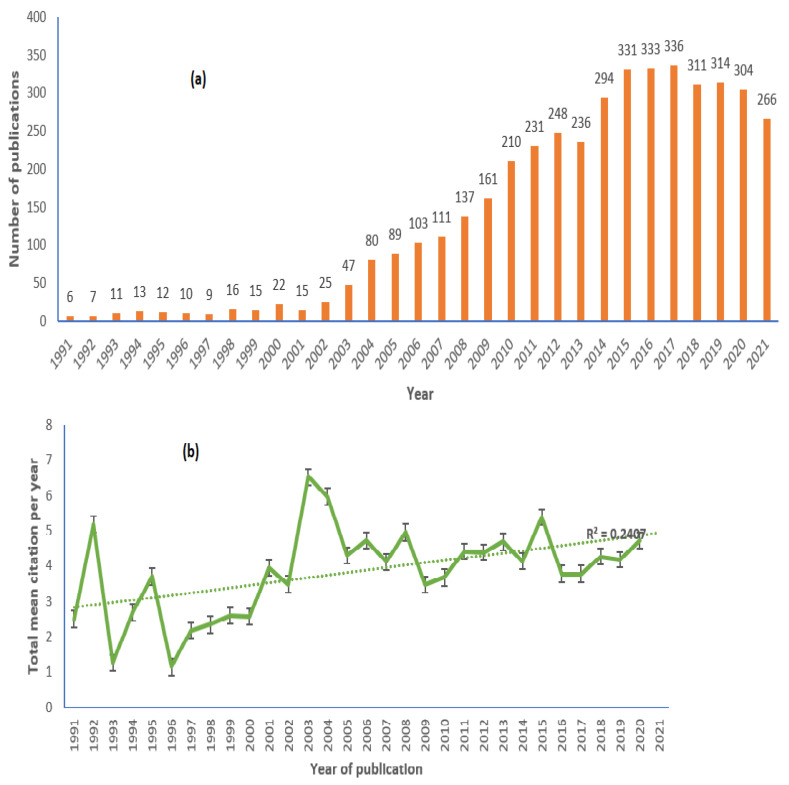
(**a**) Growth of publications on norovirus (1991–2021); (**b**) Line graphical representation of the article cited in NoV disease. The dotted line represents linear growth with R^2^ = 0.24.

Preliminary analyses were performed to observe the annual scientific research articles on norovirus disease and the average citation annually to detail the trends of research at different growth stages. Since our collection of research articles concerning articles published on norovirus disease, not many research articles have been published in this area. From 1991 to 2003, the number of published articles on NoV disease was unstable and very low. The study on norovirus disease was at an early stage, suggesting that the importance of norovirus disease did not attract the attention of most researchers. Interestingly, the published articles in NoV disease drastically increased in 2004–2012 (Figure 3a). In 2013, there was a drop in the growth of the production of research articles in NoV disease. Between 2014–2017, the number of research articles on NoV disease steadily increased. This indicates that norovirus disease has attracted the attention of many scholars from various countries. In the 2014–19 active high-performance period, records increased sharply, reaching a maximum of 336 in 2017.

Figure 3b shows the annual graphical representation of the total average citation per item. It implies the yearly average number of times NoV disease-related manuscripts have been cited. Based on the findings from this study, an increase in citations was observed in 1992 and 2014, and the lowest citations were observed in 1993 and 1996. There was an absence of stability in the evolution rate of average citations per item in the field of norovirus disease. It is noted that when a peak was reached, it declined for a few years and then recovered back to a higher peak. The research period that had the best performance among the most cited papers was between 2003 and 2017, and the maximum average citations per article were achieved at 6.51. During 2015–2017, the frequency of average citations per article proliferated to a peak of 3.78 in 2017.

The Foodborne Disease Burden Epidemiology Reference Group (FERG) was set up in 2007 by the World Health Organization (WHO) to assess the regional and global burden of all causes of food-borne illness [26]. The FERG is made up of thematic working groups responsible for assessing the human health problem of (1) enteric bacterial and viral infections, (2) parasitic infections, and (3) diseases caused by chemicals and toxins. The epidemiology of all bacterial and viral diseases potentially transmitted by food was reviewed and reported in 2015 by FERG EDTF. In addition, 22 pathogens were selected and explored for their study based on their public health significance and data availability [27]. Their studies showed that the 22 pathogens caused 2.0 billion (95% UI 1.5–3.0 billion) illnesses in 2010 and 39% (95% UI 26–53%) in children < 5 years of age. Norovirus was the leading cause of food-borne illness, accounting for 125 million (95% UI 70–251 million) cases. Among the 1.9 billion cases of diarrheal diseases, NoV was responsible for 684 million illnesses. Noroviruses cause the highest incidence of food-borne illness and the overall burden, which underscores the global importance of this agent. The etiology, risk factors and interactions of enteric infections and malnutrition and effects on children’s health and development (MAL-ED), a cohort survey of multi-site births at eight sites in South America, sub-Saharan Africa, and Asia [28] was researched by Platt et al. who estimated pathogen-specific diarrhea burdens in children aged 0–24 months at these MAL-ED study sites and reported that *Campylobacter* spp., norovirus GII, and astrovirus contribute significantly to diarrhea in children under five years of age [29].

Ettayebi and co-workers put forward a novel culture system that produces human intestinal enteroids (HIEs) from stem cells isolated from intestinal crypts in human intestinal tissues and recapitulate that the natural intestinal epithelium would support HuNoV growth [30]. These multicellular, differentiated HIEs are non-transformed, physiologically-active cultures that respond to agonists, covering multiple intestinal epithelial cell types (enterocytes, goblet, enteroendocrine, and Paneth cells). HuNoV replicates in enterocytes from diverse intestinal segments in HIEs. Moreover, the factors present in the intestinal medium, such as bile, increase or are necessary for replication. The author concluded that establishing this new cropping system would facilitate applications in several areas critical to public health, such as food security, developing new diagnostics, vaccines, therapeutics, and advanced research on the evolution of HuNoV immunity and pathogenesis.

Baldridge et al. reported that antibiotic agents prevent persistent infection with murine norovirus (MVN), an effect that has been reversed by restocking bacterial microbiota [31]. The antibiotic agents did not inhibit tissue infection or affect systemic viral replication but functioned primarily in the intestine. It concluded that the effects of antibiotics on the treatment of infectious diseases, including NoV, may not be entirely due to their antibacterial properties.

### 3.1. Historical Analysis

**Figure 4:**
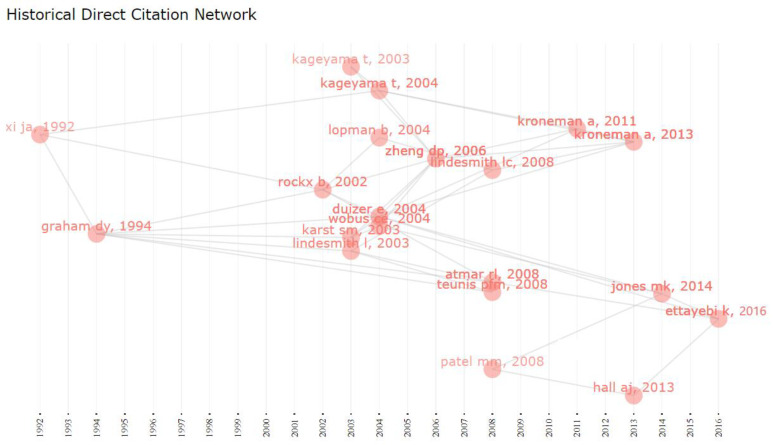
Historical analysis of direct citation of top-cited papers in the discipline of norovirus research from 1991–2021. **Note:** Each node in the figure signifies a key document, and the directional arrow specifies the citation association between the two documents.

As proposed by Garfield [32], we performed historiographic analysis, representing a sequential network map of significant immediate citations rising from a bibliographic compilation. The output, plotted with the help of histplot, showed significant works from a period being studied in their appropriate sequential context. Figure 4 provides the year-by-year mapping of the historical directly-cited papers. Figure 4 displays each node for essential articles, and the lines indicate the citation connection between the articles. There was a space between the articles’ nodes from 1995 and 2001, stating that no classically- cited articles were found during this period. The map in Figure 4 makes it visually apparent that many well-cited papers published between 2002 and 2003 were related to Xi et al. and Graham et al., which are essential in the history of NoV.

Interestingly, the author belongs to the same research group. The authors reported that Norwalk virus capsid protein was produced by expressing the open reading frames (ORF) of the Norwalk virus genome using a cell-free translation system and baculovirus recombinants [32]. By expressing one-third of the 3′ end of the Norwalk virus genome, they reported essential features of the Norwalk virus, including demonstrating that the second predicted ORF of the Norwalk virus genome encodes the viral capsid protein. In addition, they pinpoint that protein with the molecular weight of 58,000 may self-form into virus-like particles, which are immunoreactive. Additionally, the expression of the second ORF produced not only a 58,000 protein but also a 34,000 protein. It was also not ascertained with their present data whether the production and relationship between the 58 K and 34 K proteins seen in insect cells infected with the baculovirus recombinant are like those in the human intestine. They also reported that the fact that 34 K protein was immunoprecipitated by convalescent-phase serum samples signifies that the carboxy terminus of the capsid protein comprises epitopes. In addition, the availability of large quantities of recombinant Norwalk virus particles will enable fast, sensitive, and reliable tests to be developed to diagnose Norwalk virus infection and carry out structural studies.

Graham [33] et al. performed ELISAs assay using recombinant virus particles as the antigen source to evaluate virus excretion trend and individual immune responses. Additionally, they cited their previous published work. Their results showed a higher infection rate, more subclinical infections, and prolonged virus excretion following norovirus inoculation.

### 3.2. Local Citations and Global Citations from the Historical Analysis

Local citations measure the number of times an author (or document) included in this collection has been cited by documents also included in the collection. The raw articles were further examined by LCS (local citation score) and GCS (global citation score) indicators. LCS defined how many times the authors’ articles included in the collection from the WOS database have been cited by other articles in the collection. GCS defined how often the authors’ articles included in this collection have been cited, corresponding to the total citations (TC). However, the cited papers are not necessarily those in norovirus disease. Notably, the higher the LCS (Table 1), the more significant the article is on the topic of norovirus disease. The analysis detailed that the publication by Kageyama et al. [34] was among the highest LCS (530) and GCS (1043) values. The paper is titled “Broadly Reactive and Highly Sensitive Assay for Norwalk-Like Viruses Based on Real-Time Quantitative Reverse Transcription-PCR”, published in the *Journal of Clinical Virology*. The lack of a tissue culture system for norovirus propagates a significant obstacle in diagnosing norovirus infection. Kageyama et al. [34] proposed that Reverse transcription-PCR (RT-PCR) can be an attractive alternative for Norwalk virus detection [34]. The investigation of human fecal material by electron microscopy (EM) revealed different small round virus-like particles [35]. The first of these small round viruses to be discovered was the Norwalk agent defined by Kapikian et al. in 1972 [36]. However, the sensitivity of EM detection is low, requiring at least 10^6^ viral particles per mL of stool [37]. The virus was observed on samples of volunteers fed stool filtrates following an outbreak of bacteria-free gastroenteritis. Kageyama et al. [34] studied the sequences at the ORF1-ORF2 junction, the most conserved region in the Norwalk virus genome, and ascertained NLV detection assay for routine use with real-time quantitative RT-PCR. Thus, the authors consider this method worthwhile for routine diagnosis and clarifying the epidemiology of norovirus infections and, therefore, the public health control of norovirus disease.

In another study reported by Kageyama et al. [46], 416 stool specimens collected from 66 outbreaks between January 1997 and May 2002 in Saitama Prefecture, Japan, were screened by real-time RT-PCR. They analyzed the strains in each outbreak phylogenetically; the results offer a more detailed study of the molecular epidemiology of this significant public health concern. To avoid misclassifications and misperception generated from short conserved sequences and a consistent basis for NoV nomenclature. Zhang et al. analyzed 164 deduced amino acid (AA) sequences of the NoV major capsid protein, including all five genogroups present in their investigation. A distinct phylogenetic scheme of NoVs was determined, and multiple distance calculation methods examined strain clustering. The authors proposed a classification scheme for standardization of NoV nomenclature, which should aid molecular characterization and description of outbreak strains and provide genetic insights for future studies of NoV. The paper has LCS values of 477 and GCS values of 843 (Table 2), respectively. Patel et al. [39] conducted a study that used similar RT-PCR-based inclusion criteria and molecular assays to detect NoV in fecal specimens from patients with diarrhea and comprehend the etiologic role of NoV in sporadic diarrhea. Notably, the rising prevalence of NoV disease supports considering targeted interventions, such as vaccines, to reduce the extent to which young children suffer from this disease. The authors concluded that vaccines against NoV would probably be complex because immunity against these viruses and the variety and evolution of circulating strains are incomplete.

Nevertheless, genotype II, cluster 4 NoV strains acted to be the most common strains among the studies, thus may be the primary targets for vaccine development. This paper triggered two distinct citation chains. In continuing to develop cell culture for NoV, Duizer et al. [50] attempted to develop a method for the cultivation of NoV. However, cultivable NoV did not arise from the tested stool samples. Notably, the paper triggered five citations, apart from the high LCS (174) and GCS (434) values. This includes a paper titled “Mechanisms of GII.4 Norovirus Persistence in Human Populations” by Lindesmith et al. [45] published in PLOS Medicine. The authors investigated the molecular mechanisms governing GII.4 epidemiology, susceptibility, occurrence, and continuity in the human populace in this article. The outcome of their research stated that GII.4 noroviruses persisted by altering their HBGA carbohydrate complex targets. Releasing highly penetrating host susceptibility alleles allows immune-driven selection in the receptor-binding region to release protective herd immunity simultaneously. They further suggested that further studies are needed to establish whether the evolutionary patterns are distinct to the GII.4 noroviruses or epitomize a typical evolutionary pattern of the norovirus family. Their studies presented a predictive model for future empirical studies exploring the relationships among antigenic change, norovirus pathogenesis, vaccine design, and human disease. Other papers that cited Duizer et al. include Kroneman [51,52] et al. (2011 and 2013) (Proposal for a unified norovirus nomenclature and genotyping), Jones et al. [50] (Enteric bacteria promote human and mouse norovirus infection of B cells) with low LCS values of 254, 239, and 210, respectively, and high GCS value of 507, 394, and 481, respectively.

The paper “Food-borne illness acquired in the United States—major pathogens” has the highest GCS value of 1230. The reason is that the authors focused on 31 major pathogens, including NoV. The authors used data from active and passive surveillance and other sources to estimate that in the United States, 31 major pathogens were responsible for 9.4 million food-borne illness episodes, 55,961 hospitalizations, and 1351 deaths annually. Notably, NoV causes most illnesses, followed by *nontyphoidal Salmonella* spp. (11%), *Clostridium perfringens* (10%), and *Campylobacter* spp. (9%), respectively. Leading causes of hospitalization were nontyphoidal *Salmonella* spp. (35%), norovirus (26%), *Campylobacter* spp. (15%), and *Toxoplasma gondii* (8%). Meanwhile, the leading causes of death were estimated as follows: *nontyphoidal Salmonella* spp. (28%), *T. gondii* (24%), *Listeria monocytogenes* (19%), and NoV (11%).

### 3.3. Analysis of the Main Researchers

The research papers involved 4303 after removing duplicates and 15,905 authors in total, among which 4591 authors have one paper. From the standpoint of issued paper numbers (Table 3), the top 10 authors are Jang X, Vinje J, Estes MK, Hall AJ, Tan M, Atmar RL, Takeda N, Li Y, Katayama K, Ushijima H with 135 articles, 119 articles, 82 articles, 67 articles, 65 articles, 60 articles, 59 articles, 58 articles, 57 articles, and 54 articles, respectively. The American scholar, Jang X, is the first influential author regarding norovirus disease in document numbers with h-index and g-index of 45, and 76, respectively. Most of the reported papers were published in high-impact factor journals of high quality. For example, Jang X [42] published a paper titled “Human susceptibility and resistance to Norwalk virus infection” in Nature Medicine, with 688 citations. The paper titled “Norwalk virus-infection of volunteers—new insights based on improved assays” published in the *Journal of Infectious Diseases* was another outstanding achievement for Jang X [33], and the paper has 313 citations. Jang X has been publishing papers which focus on norovirus since 1991.

**Figure 5:**
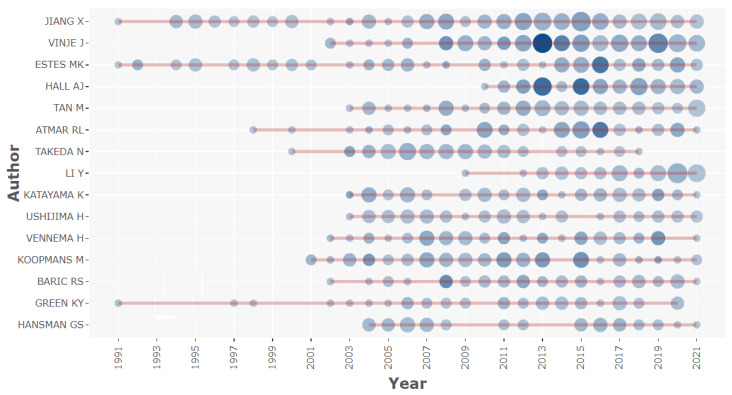
Authors’ production over time in the subject of NoV disease. **Note:** The circle size in the figure signifies the number of documents, and the shade of the color signifies the number of citations.

Interestingly, Jang X obtained the highest number of published articles and the highest frequency peak of average citations per item in 2015. The graphical representation of authors’ production over time in the subject of norovirus disease is displayed in Figure 5. The size of the circle in the figure represents the number of published articles, and color represents the number of citations.

Vinje J is another leading outstanding researcher when it comes to research on norovirus disease. The author has 119 articles; h-index and g-index of 43 and 91, respectively. Most of the reported papers from this author were published in high-impact factor journals of high quality. For example, Vinje J published a paper titled, “Enteric bacteria promote human and mouse norovirus infection of B cells” in science; the paper has 639 citations. The study of Vinje and co-workers [53] revealed that human and mouse noroviruses infected B cells in vitro and probable in vivo. Besides, several efforts to culture HuNoVs were unsuccessful, and this may be due to the nature of the cell variation tested and the lack of stimulatory carbohydrate molecules. In their investigations, reduction of mouse norovirus replication was observed in vivo when the intestinal microbiota was reduced utilizing oral antibiotic management, and concluded that intestinal B cells are in vivo targets of NoVs [53]. They identified B cells as a cellular target of noroviruses and enteric bacteria as a stimulatory factor for norovirus infection, providing an avenue for developing an in vitro infection model for human norovirus.

### 3.4. Academic Collaboration

A collaborative network is a network where nodes are authors and links are co-authors because the latter is one of the most well-researched forms of scientific collaboration. A collaborative network of authors may be obtained through:

$$\lambda =\beta^{’}\times \beta$$

**Figure 6:**
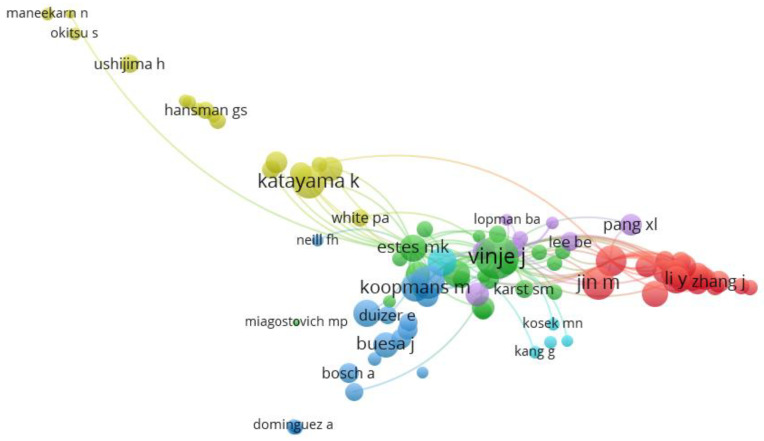
Authors’ collaboration map in the field of NoV disease. **Note:** The collaboration map of authors reflects the scientific research cooperation between them. The circle/node signifies the authors; size of the circle/node signifies the number of articles. The lines denote the authors’ collaboration strength, and each color signifies a cluster.

In this case, β is a bipartite network of manuscripts x authors and λ denotes the academic collaboration. Authors’ collaborations are part of the strength that accelerates expertise and discussion, which widens the vision of a particular subject area. As expected, there are collaborations between the authors at multiple levels in norovirus disease. VOSviewer was used to visualize the graph from the R studio software interface. Figure 6 displays circle/node, which signifies the authors, the size of the circle/node signifies the number of articles, the lines denote the strength of collaboration between the authors, and each color signifies a cluster (group of items with comparable attributes within a network) [54]. The network is positioned on Katayama K, Koopmans M, Vinje J, Jin M, Buesa J, Kosek MN, respectively. The network represented by Katayama K and Vinje J has a high clustering density, and the authors have a significant influence in the field of norovirus disease. Besides, Koopmans M and Jin M are influential authors in the subject area of norovirus disease. Koopmans M started publishing in 2001 in norovirus disease. Meanwhile, the highest number of literature outputs were recorded in 2015. Koopmans’ h-index and g-index values are 34 and 46, respectively. Koopmans M and co-workers did a collaborative network to bring together the relevant data from around Europe to focus on the increase in norovirus reports and whether the seasonal incidence pattern in 2002 changed from the previous year; most importantly, the type of NoV strains causing the recent outbreaks and why the predominant strains in 2002 differ from the preceding years [55]. The collaborative effort led to the combination of data from ten European countries, which indicated that the high increase and distinctive seasonal pattern of norovirus gastroenteritis in 2002 were due to the emergence of a new virus variant. Their results provided a reasonable virological explanation for the effect on public health.

In another reported work of Koopmans M and co-workers, different cell lines including A549, AGS, Caco-2, CCD-18, CRFK, CR-PEC, Detroit 551, Detroit 562, FRhK-4, HCT-8, HeLa, HEC, HEp-2, Ht-29, HuTu-80, I-407, IEC-6, IEC-18, Kato-3, L20B, MA104, MDBK, MDCK, RD, TMK, Vero, and 293 were evaluated in an effort to replicate NoVs [50]. Notably, none of the cell cultures effectively produced in vitro NoV replication, as determined by multiple assays. The authors concluded that phases critical for NoV replication are challenging to determine, and at least one, and likely, many phases in the replication cycle are very critical, i.e., demand specific characteristics of host cells, virus, or both.

**Figure 7:**
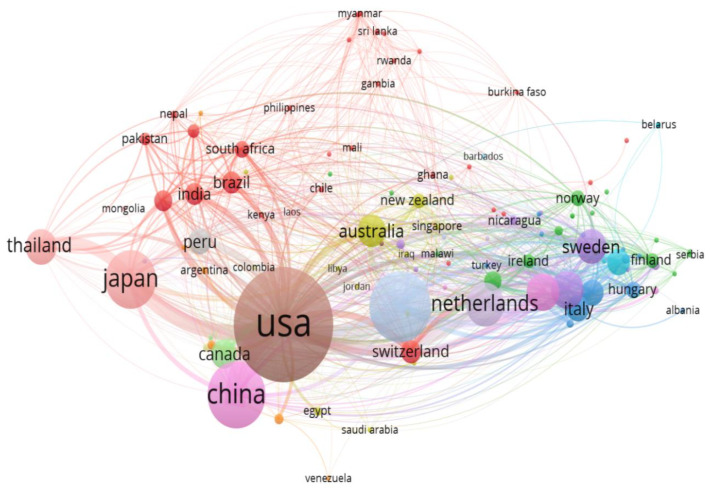
Country collaboration map in NoV research study. **Note:** The thicker the link between countries, the stronger the collaborative relationship, and vice versa.

Publishing documents in diverse countries may replicate the country’s significance and impact in the area of norovirus disease. A total of 90 countries or regions published papers between 1991 and 2021. Research output related to norovirus disease for the top 20 most active countries is shown in Table 4. Asia, North America, and Europe have the highest article productivity. This implies that developed nations play a critical role in norovirus research. Among the list of 20 countries with high productivity articles, there are five Asian countries (China, India, Japan, Korea, Thailand), one Oceania country (Australia), two North American countries (the USA and Canada), two South American countries (Brazil), and seven European countries (UK, Germany, Netherlands, Italy, France, Spain, Sweden, Finland, Belgium, Switzerland, and Denmark). Notably, no African can make the top 20 countries. The United States has an excellent performance in international collaboration and a high number of published articles (n = 1185, 27.6%) in the field of norovirus disease. Most importantly, financial intensity, availability, accessibility of research resources, and funding allowed the USA to emerge as the first country in articles’ productivity. Including a high level of intranational and potentially multinational collaboration with other institutions can affect the visibility of research and the frequency of citations [56]. As indicated in Figure 7, their frequent collaborators’ countries include China, Canada, Italy, Japan, the United Kingdom, Germany, Korea, and the Netherlands. The USA came out ahead in terms of citations (n = 55,528), followed by the United Kingdom (n = 10,736), and the Netherlands (n = 10,392). The frequency of publication varied according to the most prominent countries, from 0.8 to 27.6%. Other countries which were the top 20 according to the country citation were Japan (n = 9689), China (n = 5629), Germany (n = 5319), France (n = 4695), Australia (n = 4301), Sweden (n = 3342), and Italy (n = 3025). Notably, African countries are missing in the top 20 list based on the productivity of the number of articles and most cited countries. The VOSviewer software was employed to produce a collaboration network map of the countries from the R studio interface. Moreover, each node denotes a country; the thicker the link flanked by the countries, the stronger the pooled connections, and vice versa. Interestingly, most countries relate to lines, as shown in Figure 7, whereas the lines signify a collaboration network between the countries.

### 3.5. Multiple Correspondence Analysis and Cluster Analysis of High-Frequency Keywords

**Figure 8:**
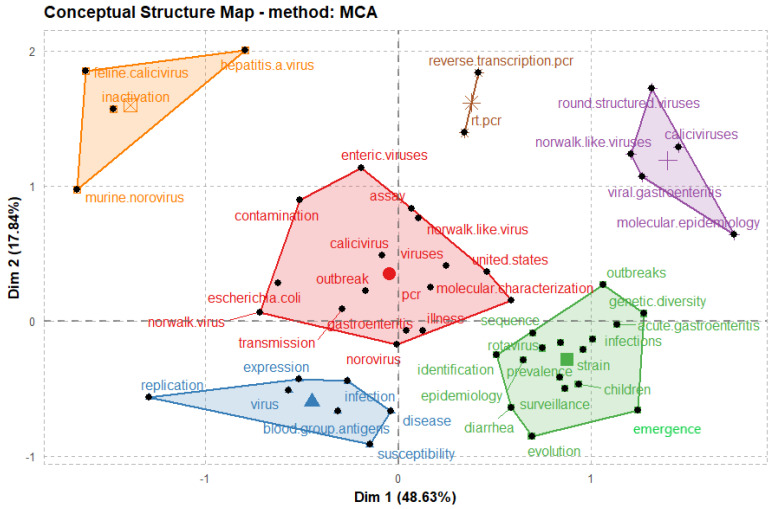
Multiple correspondence analysis of high-frequency keywords in NoV disease research articles. **Note:** The figure shows that the cluster closest to the center is the core cluster.

**Figure 9:**
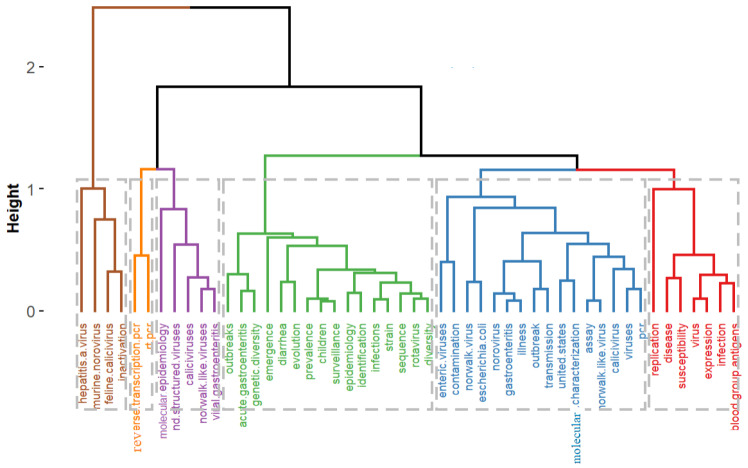
Dendrogram of hierarchical cluster analysis of author key words. **Note:** The entire classification structure forms a tree dendrogram, displaying the close association between the keywords in the field of NoV disease.

Multiple correspondence analysis (MCA) was utilized in this study to analyze and detail the keywords that are common to the articles. MCA is a descriptive method that evaluates simple two-dimensional and multiway tables containing correspondence measures between rows and columns [57]. In addition, MCA’s output produces two-point clouds typically represented by a 2-dimensional graph, and the cloud of keywords is constructed on distances between keywords [58]. The graphical potential of MCA synopses the expression of relations between the articles with no underlying hypotheses. The indicator levels that share similar characteristics are located close together and were well indicated in a 2-dimensional plot forming points clouds. The closer the articles’ keywords are to each other, the more they are related (Figure 8). As shown in Figure 9, hierarchical clustering was utilized to cluster the keywords; the other two clusters with the uppermost similarity scale were clustered to generate another cluster. After which, a tree dendrogram that detailed the correlation and decorrelation between the keywords is generated. The plot revealed six clusters represented as cluster 1 (red), cluster 2 (light blue), cluster 3 (green), cluster 4 (purple), cluster 5 (orange), and cluster 6 (brown).

Considering the findings from Figure 8 and Figure 9, the research articles on NoV disease can be abridged into the following categories as follows:

***Cluster 1:*** The first cluster is mainly on the studies conducted to estimate the countries’ health burden by focusing on different pathogens, including NoV. The published work of Platts-mill et al. [29] reported the pathogen-specific burdens of community diarrhea in developing countries. Children within 17 days of birth at different locations including Dhaka, Venda, South Africa, and Haydom, Tanzania, Bangladesh, Fortaleza, and Brazil, Vellore, India, Bhaktapur, Nepal, Loreto, Peru, Naushero Feroze, Pakistan, were registered and monitored using qualitative diagnostics to evaluate a subset of samples. A total of 2145 children were enrolled, among which 1740 have follow-up data for 2 years. Notably, among the sites and incidents, the highest burden of diarrhea was caused by norovirus GII, astrovirus, rotavirus, *Campylobacter* spp., and *Cryptosporidium* spp. in the first year of life, and followed by *Campylobacter* spp., norovirus GII, rotavirus, astrovirus, and *Shigella* spp. in the second year of life. Norovirus GII appears to have contributed to diarrhea incidence at several sites. The study of Platts-mill et al. on the diversity of pathogens associated with community diarrhea in children in low-income and middle-income countries varied between sites. High rates of enteropathogens were spotted in non-diarrhea samples. Hence, the authors proposed that *Campylobacter* spp., norovirus GII, and astrovirus contribute significantly to the burden of diarrhea in children. In continuing their work, the samples used previously in their study were retested using quantitative PCR to improve the etiology estimates. They were tested for a broader range of pathogens and identified clinical characteristics that help differentiate between various causes, guide the treatment algorithms, and improve antibiotic use for children with diarrhea in low-resource settings [59]. NoV was among the ten pathogens that accounted for 95.7% of attributable diarrhea in the reported results. Other pathogens include *Shigella*, sapovirus, rotavirus, adenovirus, enterotoxigenic *Escherichia coli*, astrovirus, *Campylobacter jejuni* or *C coli*, *Cryptosporidium,* and typical enteropathogenic *E coli*. The authors successfully used quantitative molecular diagnostics to identify the ten pathogens responsible for most community-based childhood infectious diarrhea in diverse low-resource settings. These include several pathogens for which their burdens have been previously substantially underestimated.

***Cluster 2:*** Norovirus outbreaks, a food- or waterborne transmission, is the most common source of prevalent disease, and they can stay for long periods in contaminated food and act to survive various food processing and storage conditions. Enzyme immunoassays and reverse transcription-polymerase chain reaction (RT-PCR) are presently used to detect NoV. RT-PCR remains the gold standard for detecting NoV compared to enzyme immunoassays [60]. Nenonen and colleagues reported the virological evaluation of an extensive non-seasonal public outbreak of acute nonbacterial gastroenteritis. Including strong epidemiologic indices of waterborne contamination of a municipal water supply [61]. The authors showed that NoV exhibits remarkable genomic diversity that challenges molecular diagnostic methods. Real-time RT-PCR revealed strains with 26% amino acid dissimilarity to established genotypes, which is criteria for defining a new genotype. The strain diversity detected in this waterborne outbreak emphasizes the importance of extended molecular surveillance of non-cultivable NoVs. Mormann and co-workers stated that it was uncertain whether the precautions taken in industrial food processing for preservation and processes can inactivate NV in contaminated foods [62]. As a risk evaluation method, Mormann and co-workers experimented on contaminated foods belonging to different products with a defined number of infective human NV GGII. They further subjected the food to physicochemical evaluations such as freezing, cooling, acidification, and heating. With the help of real-time RT-PCR, viral genome copies were quantified, and thus measured the actual reduction in the number of intact NV particles. The methods established and data obtained in their reported work could benefit process optimization for NV inactivation in foods and promote the development of risk assessment systems to improve consumers’ protection.

***Cluster 3–5:*** The third cluster is mainly on the genogroups and genotypes that initiate most norovirus-related gastroenteritis outbreaks worldwide. Notably, understanding the molecular epidemiology of norovirus genotypes is essential considering the advancement of vaccines under preclinical studies and clinical trials. Currently, there is a dark indication of whether the vaccine formulations will effectively inhibit genogroups and genotypes viruses that are primarily associated with norovirus outbreaks and hospitalizations [63,64,65]. Burke and co-workers reported identifying settings, spread routes, seasonality, warning signs, and patient demographics related to norovirus genotypes [66]. Additionally, the authors enumerate the associations between these attributes and hospitalizations and deaths from the norovirus outbreaks. The authors analyzed the dataset of the National Outbreak Reporting System (NORS) in 2009–2016 of acute norovirus outbreaks linked to laboratory-confirmed norovirus outbreaks reported to CaliciNet to detail the differences in genotypes (GII.4 vs. non-GII.4). A total of 3747 outbreaks (53.1% of CaliciNet outbreaks and 24.8% of NORS outbreaks) were related between the two systems. Their results showed that GII.4 strains were reported in 2353 (62.8%) of outbreaks; the most reported non-GII.4 genotypes include GI.3 (n = 229 (6.1%)), GII.6 (n = 214 (5.7%)), and GII.2 (n = 172 (4.6%)). In addition, GII.4 norovirus outbreaks occurred more commonly in healthcare settings, affected people of old age, were more likely to occur during the typical norovirus season (November–April), and were significantly more likely to be associated with transmission food. They concluded that GII.4 viruses should be included in norovirus vaccines under development to reduce hospitalizations and mortality due to norovirus outbreaks. People in the healthcare environment should be deemed potential targets. Notably, the health providers should be alert to the possibility of more severe outcomes when a new variant of GII.4 emerges.

In another study reported by Ferreira et al. [67], the effect of NoV as a causative agent of acute gastroenteritis in both outpatients and inpatients in the State of Rio de Janeiro for the period of a 4-year surveillance program was demonstrated. A total of 1687 fecal specimens were obtained from gastroenteritis patients; meanwhile, 324 were rotavirus-positive, 1363 rotavirus-negative, and 1087 samples were tested for NoV RNA. Besides, 267 outpatients from Municipal Public Health Centers and 820 inpatients whose samples were obtained by active surveillance in Public Hospitals were enrolled. Reverse transcription (RT) and polymerase chain reaction (PCR) detected NoV GI and GII in the fecal samples. The results showed 35.1% (381/1087) positive samples for NoV, comprising of 30.2% (248/820) from inpatients and 49.8% (133/267) from outpatients, respectively. Unlike the report of Burke and co-workers on seasonal patterns in NoV infection, Ferreira et al. observed a lack of seasonality in NoV infection in patients admitted to hospital. However, two spikes of NoV infections were detected in outpatient cases, suggesting that outbreaks occurred during these periods. Notably, the molecular classification revealed GII as the predominant genogroup, 96.3% (104/108) of all sequences analyzed, and GII.4 was the most common genotype detected (80.7%). This result corroborated with the molecular epidemiology of norovirus genotypes reported by Burke and co-workers [66]. Another genotype and genogroup observed include GII.6, 3, 14, 7, and 8, GI.2, and GI.3. Notably, the authors concluded that implementing national and international surveillance programs for NoV infection through a network database can assist in monitoring the acute gastroenteritis outbreaks and help shed light on pathways of transmission and sources of NoV infection. Although the GII.4 is the cause of the most norovirus outbreaks, GII.17 is another variant that has been a menace to humankind. GII.17 may soon substitute GII.4 as the leading cause of norovirus outbreaks globally based on some epidemiological evidence [68,69,70,71,72,73]. The study of Yi et al. [74] produced a panel of anti-GII.17 mAbs, and through an experiment map, a blockade epitope in GII.17 noroviruses’ capsid protein for the first time. Hence, reporting the evolution and persistence of GII.17 in humans as well as information is essential for future development of norovirus vaccines.

***Cluster 6:*** The development of antiviral drugs targeting HuNoV has been elusive due to the lack of a cell culture system for HuNoV. Feline calicivirus (FCV) and murine norovirus (MNV), family *Caliciviridae*, have similar physical characteristics, genome organization, and replication strategies to those of human NoV [75,76,77]. However, FCV belongs to the genus Vesivirus. This is different from the genus of HuNoV and has been used as a surrogate virus for HuNoV [41]. In addition, MNV is noted as an effective surrogate for HuNoV among cultivable viruses since it belongs to the same genus and is most like HuNoV [78]. The earliest MNV was revealed by Karst et al. in 2003, followed by reporting of many other MNV strains [43]. Notably, Cluster 1–6 has provided the research article about the epidemiology, pathogenicity, evolution, and persistence of diverse norovirus genotypes and different norovirus databases. Identifying the essential characteristics of NoV will be critical in developing a longstanding approach for reducing the burden of NoV and in preventing these disease epidemics in a rapidly evolving world.

### 3.6. Thematic Evolution Analysis

**Figure 10:**
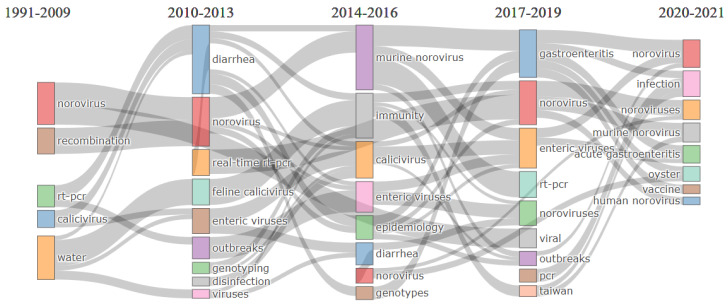
Thematic evolution of keywords in field of research on NoV disease 1991–2021. **Note:** This thematic helps analyze the flow conditions of diverse themes in the field of NoV, and clarifies quantitative evidence such as thematic flow, direction of thematic flow, and conversion associations. The thicker the line, the higher the significance of the two themes over the year. Color helps distinguish diverse research themes.

The thematic evolution detailed the varying evolutionary associations that displayed field development and saw the point of development direction, evolution routes, and evolutionary drifts of the research field’s thematic content, intensity, and structures [54,79]. The main objective is for the thematic detection and identification of essential topics between 1991–2021, including thematic change and evolution in the norovirus research field. Sankey diagram is used to interpret the thematic evolution results, and to visualize and categorize the detected themes of the studied field. Each node in the Sankey chart signifies a subject, and node size is commensurate with the number of keywords included in the subject. Meanwhile, the line connections between nodes represent the research topic’s evolving focus. The width indicates the number of shared keywords; the more comprehensive the line, the greater the significance of the two subjects. The period of 30 years considered for our collection of articles was split into five periods: 1991–2009, 2010–2013, 2014–2016, 2017–2019, and 2020–2021, as shown in Figure 10. The thematic evolution was analyzed using the authors’ keywords. In the first period, 1991–2001, thematic evolution is observed only in five research areas: norovirus, recombination, RT-PCR, calicivirus as basic theme, and water as a motor theme. Moreover, the second period, 2010–2013, showed real-time RT-PCR, and evolved as an emerging theme while norovirus remains as the primary theme and seven new thematic areas emerged: diarrhea, feline calicivirus, enteric viruses, outbreak, genotyping, disinfection, viruses. This suggests that the number of articles on norovirus diseases has progressively increased over time, and the thematic evolution has been ongoing.

Direct and immune electron microscopy (EM) has been a valuable tool in detecting norovirus in fecal specimens since EM first detected rotavirus in fecal specimens. However, the laboratory’s low sensitivity and lack of implementation because of technical drawbacks and reliance on qualified medical personnel for its operation have disadvantaged it. Other methods include radioimmunoassay (RIA), enzyme immunoassay (EIA), real-time reverse transcription-polymerase chain reaction (RT-PCR), and next-generation sequencing (NGS). Subsequently, RT-PCR assays are currently the best method for detecting norovirus due to their unrivaled sensitivity and ability to detect genetically different NoV strains [80]. Neesanant et al. [81] concluded this in their work after optimizing one-step real-time RT-PCR assays established to detect NoVs accurately and specifically from stool specimens. However, Siebenga and co-workers [82] proposed that population immunity is crucial in the epochal evolution of novel norovirus strains, one of the determining factors for novel epidemic strains. Neesanant et al. believed that identifying the circulating strains’ population and acquiring knowledge about the evolution of NoV will be helpful to identify vaccine components as new vaccine candidates are tested. The authors also reported that the assays are quick, practical, and highly reactive to a broad spectrum of NoV genotypes and should be an effective tool for surveillance and epidemiology. In the study of Jiang et al. [83] titled “Detection of Norwalk Virus in Stool by Polymerase Chain Reaction”, it was detailed that RT-PCR assays will be suitable for detecting Norwalk virus in food or environmental food samples such as shellfish and shellfish waters. Quantitative RT-PCR for the enumeration of noroviruses (Norwalk-like viruses) in water and sewage was reported by Laverick et al. [84]. The authors proposed that quantitative determination of noroviruses in water will pave the way for in-depth analysis of water, foodstuffs, and other matrices, and assessed the risk posed by this organism in the environment.

In the third period, 2014–2016, murine norovirus, immunity, calicivirus, enteric viruses, epidemiology, diarrhea, norovirus, and genotypes evolved in the thematic evolution. The development of antiviral agents and vaccines for HuNoV has been delayed due to the lack of cell culture. Since HuNoV cannot be cultivated in a cell culture system, NoV surrogates are used to investigate the efficacy of chemical disinfectants [85]. The authors investigated the pre-, co-, and post-treatment of Korea red ginseng (KRG) extract and ginsenosides on norovirus surrogates, namely MNV and FCV. Subsequently, the pre-treatment with KRG and ginsenoside Rg1 and Rb1 significantly inhibit FCV and MNV dose-dependently. Unlike co-treatment or post-treatment, KRG and the ginsenosides were ineffective at inhibiting FCV and MNV in vitro models.

Meanwhile, the authors proposed that because some ginsenosides inhibit the virus by enhancing humoral and cellular immunity, they could excellently impede NoV in vivo. There is still a need to elucidate the antiviral mechanisms of KRG and ginsenosides, and identify the active compounds in further studies. Arias and colleagues [86] investigated the anti-norovirus activity of favipiravir.

Interestingly, favipiravir as a nucleoside decreases viral load in vivo by employing antiviral mutation in a mouse model for norovirus infection and providing evidence for the use of favipiravir derivatives or mutagenic nucleosides in the clinical treatment of noroviruses during an outbreak, especially in enclosed environments such as hospitals or nurseries. Despite the low inhibitory activity of 2CMC (2′-C-methylcytidine: antiviral therapy for HCV) in vitro in MNV replication, the compound excellently decreases virus shedding in the feces of mice chronically diseased with MNV compared to favipiravir without the emergence of drug resistance [87]. The paper title “Prophylactic treatment with the nucleoside analogue 2CMC completely prevents transmission of norovirus” was reported by Rocha and co-workers [88]. The study showed that a small-molecule inhibitor of norovirus replication could effectively inhibit the transmission of MNV from infected to uninfected mice, acting as an essential tool to control norovirus outbreaks, subsequently, protecting uninfected people against the norovirus infection and treatment of chronically infected immunocompromised patients. Rocha and colleagues also reported inhibition of human norovirus by a virus polymerase inhibitor in the B-cell culture system of the mouse model [89].

The inclusion of post-exposure antiviral treatment against norovirus infections effectively protects against diarrhea and reduces viral excretion in the feces in mouse mortality mode [90]. Notably, 2CMC has been tested in every available model and considered a reference compound for NoV [91]. Ribavirin is an analogue of guanosine and the first drug that inhibits MNV and HuNoV replication [92]. CMX521 is a novel nucleoside antiviral drug discovered by the Chimerix and is presently in phase I clinical trials with potent antiviral activity against NoV [93]. Structure-guided design and optimization of dipeptidyl inhibitors of norovirus 3CL protease, and animal studies using the mouse model of MNV infection has been reported [94]. Among the reported compounds, 3-Chlorobenzyl ((S)-3-Cyclohexyl-1-oxo-1-(((S)-1-oxo-3-((S)-2-oxopyrrolidin-3-yl)propan-2-yl)amino)propan-2-yl)carbamate was found to be superior with an EC_50_ value of 80 nM, and significantly reduced the virus titers in the small and large intestines at three days post virus infection by 42.12- and 7.98-fold, respectively, compared to the untreated control group. Findings of the study pinpoints a non-nucleoside compound that exhibits effectiveness in the murine model of norovirus infection and, thus, are appropriate for performing further preclinical studies. Other non-nucleosides include PPNDS, suramin, and NF023 [95,96,97]. These compounds have not reached clinical trials due to poor cell permeability [93].

Furthermore, the fourth period showed gastroenteritis, norovirus, enteric viruses, RT-PCR, noroviruses, viral, outbreak, PCR, and Taiwan (2017–2019). At the same time, the fifth period showed norovirus, infection, noroviruses, murine noroviruses, acute gastroenteritis, oyster, vaccine, and HuNoV (2020–2021), respectively. Developing an effective anti-norovirus vaccine has been a challenge; thematic evolution showed that research articles on the vaccine for norovirus were more pronounced in the past few years. In an attempt to develop a norovirus vaccine, Wang et al. investigated the possibility of formulating a combination vaccine comprising two different virus-like particles (VLPs) derived from enterovirus 71 (EV71) and NoV GII.4 [98]. Their result showed the bivalent vaccine-elicited durable antibody responses toward GII.4 and EV71. There is no immunological interference between the EV71- and GII.4-VLPs, thus substantiating further preclinical and clinical development of a novel VLP-based bivalent vaccine for EV71 and NoV GII.4. The authors also reported the paper titled, “Development of a Surrogate Neutralization Assay for Norovirus Vaccine Evaluation at the Cellular Level.” Other research on vaccines includes intranasal delivery of a bivalent norovirus vaccine formulated in an in situ gelling dry powder [99], immunogenicity in a Phase 1 trial in healthy adults [100], multivalent P particle-based vaccines [101], polyvalent norovirus P domain–GST complexes [102], and monovalent intranasal vaccine containing a GI.1 VLP [103]. Notably, it is detailed from the thematic evolution analysis that adequate animal models and cell culture systems are essential for evaluating the effectiveness of vaccines and antivirals. There is still a need to develop effective vaccines and antiviral drugs with minimal side effects that can be used against a broad spectrum of norovirus strains. In addition, the thematic map has allowed us to obtain more understanding of the field’s status and its future sustainability. This will also help inform researchers and stakeholders about the potentials of future research development of thematic areas within the field of norovirus disease.

**Figure 11:**
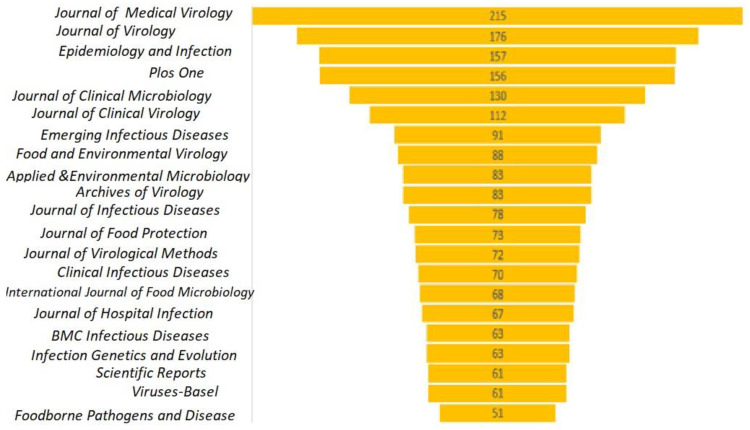
Top 21 journals with NoV articles published between 1991–2021. **Note:** The numbers represent the article count.

The research articles were analyzed based on journal sources during 1991–2021 for researchers to identify a reliable reference and when searching for journals to publish their research manuscripts. The top 21 journals with the most norovirus-related articles published are presented in Figure 11. Among the journals in which norovirus-related articles were published, the *Journal of Medical Virology* has published the most, followed by the *Journal of Virology*. In addition, the research articles in the *Journal of Virology* have total citations of 11,185, h index of 64, and g index of 98. Other core journals include *Epidemiology* and *Infection, Plos One*, and *Journal of Clinical Microbiology*. The study of norovirus disease may be concluded to include multidisciplinary research, such as virology, epidemiology, environmental science, chemistry, immunology, and microbiology.

### 3.7. Funding Analysis

The funding agencies for norovirus disease are mapped out by performing the funding analysis. The documents were excluded from the funding analysis if the authors did not mention funding sources in the paper or reported that no funding was received. The top funding agencies include: United States Department of Health Human Services (834 articles), National Institutes of Health USA (747 articles), National Institute of Allergy Infectious Diseases (501 articles), European Commission (301 articles), Ministry Of Education Culture Sports Science and Technology Japan (164 articles), National Natural Science Foundation of China (148 articles), Japan Society for the Promotion of Science (137), National Institute Of Diabetes Digestive Kidney Diseases (126), Welcome Trust (114 articles), Grants in Aid for Scientific Research Kakenhi (101 articles), Centers for Disease Control Prevention USA (96 articles), UK Research Innovation (96 articles), National Institute Of General Medical Sciences (82 articles), National Cancer Institute (80), National Center for Research Resources (77 articles), Ministry of Health Labour and Welfare Japan (73 articles), Eunice Kennedy Shriver National Institute of Child Health Human Development (65 articles), United States Department of Agriculture (62 articles), and Conselho Nacional De Desenvolvimento Cientifico E Tecnologico Cnpq (60 articles). The United States and Europe remain essential sources of publications and funding; therefore, it was not surprising that the United States has the most significant number of publications.

### 3.8. Strengths and Limitations

The present study presents some strong points compared to previous studies that adopted only systematic reviews. Foremost a strength is that it is the first bibliometric analysis study to our knowledge that embodies published articles on norovirus from 1991–2021. Another strength is examining the quality of publications included in the bibliometric analysis. Furthermore, by searching the Web of Science for the complete data source, the research team compiled a comprehensive list of the most cited documents, journals, and countries. However, an insignificant number of publications might be in other related data sources, but our findings can contribute to knowledge and indicate future research. Hence, we will consider including other data sources as additional publication sources as part of the future direction.

## 4. Conclusions

The current study examined and assessed the global scientific production in the NoV research, analyzing records assembled in Web of Science and identifying the best researchers currently available, mapping their geographic distribution, and publishing journals. This is the first bibliometric analysis to evaluate NoV research activity. The results showed a constant increase in NoV research publications over the last three decades. While our analysis indicates that scientists are still actively exploring the unexplored field of norovirus disease, fewer researchers are participating in norovirus disease studies than in other viral studies worldwide. We hope that this bibliometric analysis provides a valuable reference on critical issues and future trends in norovirus research, not only for researchers who have been involved in this area but also for new scientists who are getting ready to become active participants in this area. In addition, the analysis offers intuitions into scientific research, which will help make evidence-based descriptions and visualize research output in NoV research. It can also be used to elucidate patterns of presentation and the impact of NoV research. We expect to see further advances in developing a vaccine and an antiviral against norovirus disease targeting a broad spectrum of genotypes in the future. The results of this study are expected to further the worldwide NoV research agenda, and policymakers could use the results to strengthen investment policies in NoV R&D.

## Figures and Tables

**Table 1:** Local citations from the historical analysis.

Articles	DOI	Year	LCS	GCS
KAGEYAMA T, 2003, J CLIN MICROBIOL [34]	10.1128/JCM.41.4.1548-1557.2003	2003	530	1043
ZHENG DP, 2006, VIROLOGY [38]	10.1016/j.virol.2005.11.015	2006	477	843
PATEL MM, 2008, EMERG INFECT DIS [39]	10.3201/eid1408.071114	2008	446	697
TEUNIS PFM, 2008, J MED VIROL [40]	10.1002/jmv.21237	2008	354	794
WOBUS CE, 2004, PLOS BIOL [41]	10.1371/journal.pbio.0020432	2004	335	624
LINDESMITH L, 2003, NAT MED [42]	10.1038/nm860	2003	305	688
KARST SM, 2003, SCIENCE [43]	10.1126/science.1077905	2003	295	584
ATMAR RL, 2008, EMERG INFECT DIS [44]	10.3201/eid1410.080117	2008	270	478
XI JA, 1992, J VIROL [32]	10.1128/JVI.66.11.6527-6532.1992	1992	258	652
LINDESMITH LC, 2008, PLOS MED [45]	10.1371/journal.pmed.0050031	2008	256	407

**Note:** GCS: global citation score; LCS: local citation score.

**Table 2:** Global citations from the historical analysis.

Articles	DOI	Year	LCS	GCS
SCALLAN E, 2011, EMERG INFECT DIS [47]	10.3201/eid1701.P11101	2011	67	1230
KAGEYAMA T, 2003, J CLIN MICROBIOL [34]	10.1128/JCM.41.4.1548-1557.2003	2003	530	1043
ZHENG DP, 2006, VIROLOGY [38]	10.1016/j.virol.2005.11.015	2006	477	843
TEUNIS PFM, 2008, J MED VIROL [40]	10.1002/jmv.21237	2008	354	794
PATEL MM, 2008, EMERG INFECT DIS [39]	10.3201/eid1408.071114	2008	446	697
KULLDORFF M, 2005, PLOS MED [48]	10.1371/journal.pmed.0020059	2005	2	689
LINDESMITH L, 2003, NAT MED [42]	10.1038/nm860	2003	305	688
XI JA, 1992, J VIROL [32]	10.1128/JVI.66.11.6527-6532.1992	1992	258	652
ETTAYEBI K, 2016, SCIENCE [30]	10.1126/science.aaf5211	2016	210	639
CADWELL K, 2010, CELL [49]	10.1016/j.cell.2010.05.009	2010	49	628

**Note:** GCS: global citation score; LCS: local citation score.

**Table 3:** Top 20 influential authors in the field of NoV disease.

Authors	Articles	h Index	g Index	Publication Year Start
Jiang X	135	45	76	1991
Vinje J	119	43	91	2002
Estes MK	82	36	76	1991
Hall AJ	67	27	64	2010
Tan M	65	30	54	2003
Atmar RL	60	30	60	1998
Takeda N	59	27	58	2000
Li Y	58	12	23	2009
Katayama K	57	29	55	2003
Ushijima H	54	24	38	2003
Vennema H	51	33	51	2002
Koopmans M	46	34	46	2002
Baric RS	45	28	44	2002
Green KY	45	26	44	1991
Hansman GS	44	26	43	2004
Wobus CE	44	23	44	2003
Xia M	44	23	38	2007
Lopman BA	42	21	40	2003
Vesikari T	39	19	30	1999
Oka T	38	25	38	2004

**Table 4:** Productivity based on number of articles at country level.

	Productivity Based on No. of Articles						Most Cited Countries		
Entry	Country	Articles	Freq	SCP	MCP	MCP_Ratio	Country	TC	AAC
1	USA	1185	27.6	913	272	0.2295	USA	55,528	46.86
2	CHINA	421	9.8	334	87	0.2067	UNITED KINGDOM	10,736	36.64
3	JAPAN	369	8.6	267	102	0.2764	NETHERLANDS	10,392	71.18
4	UNITED KINGDOM	293	6.8	193	100	0.3413	JAPAN	9689	26.26
5	GERMANY	197	4.6	153	44	0.2234	CHINA	5629	13.37
6	KOREA	165	3.9	151	14	0.0848	GERMANY	5319	27
7	NETHERLANDS	146	3.4	92	54	0.3699	FRANCE	4695	36.4
8	ITALY	131	3.1	103	28	0.2137	AUSTRALIA	4301	35.55
9	FRANCE	129	3	91	38	0.2946	SWEDEN	3342	32.76
10	AUSTRALIA	121	2.8	91	30	0.2479	ITALY	3025	23.09
11	BRAZIL	109	2.5	88	21	0.1927	KOREA	2456	14.88
12	SPAIN	105	2.5	82	23	0.219	FINLAND	2405	27.97
13	SWEDEN	102	2.4	58	44	0.4314	CANADA	2320	24.95
14	CANADA	93	2.2	72	21	0.2258	SPAIN	2061	19.63
15	FINLAND	86	2	72	14	0.1628	BRAZIL	1449	13.29
16	BELGIUM	56	1.3	30	26	0.4643	BELGIUM	1163	20.77
17	SWITZERLAND	37	0.8	23	14	0.3784	SWITZERLAND	1112	30.05
18	INDIA	35	0.8	21	14	0.4	NEW ZEALAND	1014	37.56
19	DENMARK	32	0.8	17	15	0.4688	DENMARK	806	25.19
20	THAILAND	32	0.8	13	19	0.5938	INDIA	703	20.09

SCP: single country publications, MCP: multiple country publications, TC: total citations, AAC: average article citations.

## Data Availability

Raw and processed data are available upon request to the corresponding author.
